# Single-cell characterization of macrophages in glioblastoma reveals MARCO as a mesenchymal pro-tumor marker

**DOI:** 10.1186/s13073-021-00906-x

**Published:** 2021-05-19

**Authors:** Andrew X. Chen, Robyn D. Gartrell, Junfei Zhao, Pavan S. Upadhyayula, Wenting Zhao, Jinzhou Yuan, Hanna E. Minns, Athanassios Dovas, Jeffrey N. Bruce, Anna Lasorella, Antonio Iavarone, Peter Canoll, Peter A. Sims, Raul Rabadan

**Affiliations:** 1 Department of Systems Biology, Columbia University Irving Medical Center, New York, NY USA; 2 Program for Mathematical Genomics, Columbia University Irving Medical Center, New York, NY USA; 3 Department of Pediatrics, Columbia University Irving Medical Center, New York, NY USA; 4 Department of Biomedical Informatics, Columbia University Irving Medical Center, New York, NY USA; 5 Department of Neurological Surgery, Columbia University Irving Medical Center, New York, NY USA; 6 Department of Pathology & Cell Biology, Columbia University Irving Medical Center, New York, NY USA; 7 Institute for Cancer Genetics, Columbia University Irving Medical Center, New York, NY USA; 8 Department of Neurology, Columbia University Irving Medical Center, New York, NY USA; 9 Department of Biochemistry & Molecular Biophysics, Columbia University Irving Medical Center, New York, NY USA

**Keywords:** Glioblastoma, Single-cell RNA-seq, Cancer immunotherapy, Macrophages

## Abstract

**Background:**

Macrophages are the most common infiltrating immune cells in gliomas and play a wide variety of pro-tumor and anti-tumor roles. However, the different subpopulations of macrophages and their effects on the tumor microenvironment remain poorly understood.

**Methods:**

We combined new and previously published single-cell RNA-seq data from 98,015 single cells from a total of 66 gliomas to profile 19,331 individual macrophages.

**Results:**

Unsupervised clustering revealed a pro-tumor subpopulation of bone marrow-derived macrophages characterized by the scavenger receptor MARCO, which is almost exclusively found in IDH1-wild-type glioblastomas. Previous studies have implicated MARCO as an unfavorable marker in melanoma and non-small cell lung cancer; here, we find that bulk MARCO expression is associated with worse prognosis and mesenchymal subtype. Furthermore, MARCO expression is significantly altered over the course of treatment with anti-PD1 checkpoint inhibitors in a response-dependent manner, which we validate with immunofluorescence imaging.

**Conclusions:**

These findings illustrate a novel macrophage subpopulation that drives tumor progression in glioblastomas and suggest potential therapeutic targets to prevent their recruitment.

**Supplementary Information:**

The online version contains supplementary material available at 10.1186/s13073-021-00906-x.

## Background

Glioblastoma (GBM) is a devastating primary brain malignancy. Recurrence of GBM is inevitable despite the standard treatment of surgery, chemotherapy, and radiation—and the median survival is limited to around 16 months [1]. Barriers to treatment include the complex interactions of macrophages in the tumor microenvironment, which play a variety of pro-tumor roles in gliomas [2–4]. While immunotherapies have been successful across a variety of other cancers [5], they are hindered in GBM by an immunosuppressive microenvironment including tumor-associated macrophages (TAMs) [6]. Indeed, a recent study of checkpoint inhibitor therapy in GBM found an association between infiltration of HLA class II-deficient macrophages with tumor profiles unfavorable to immunotherapy [7]. In addition, these TAMs are also hypothesized to contribute to the mesenchymal expression subtype, traditionally associated with poorer outcomes and treatment resistance [8].

Targeting these TAMs (such as with CSF1R inhibitors) is an intriguing therapeutic option but requires a better understanding of markers specific to and necessary for TAM functioning [9–11]. Our classical knowledge of macrophage polarization (M1 vs. M2) provides a simplified illustration of these different states [4]. However, this is clearly not the whole picture, given that M1 and M2 genes are often co-expressed in individual TAMs [12]. Furthermore, in GBMs, there is a diverse spectrum of monocytic lineage cells (which we broadly term as “macrophages”), comprising bone marrow-derived macrophages (BMDMs) recruited from the blood as well as tissue-resident microglia. Thus, the specific markers and pathways involved in pro-tumor macrophages in GBM still remain elusive.

Due to the diversity of cell types in the tumor microenvironment, bulk expression profiles are not ideal for investigating cellular subpopulations. Instead, single-cell RNA sequencing (scRNA-seq) has proven instrumental in understanding the heterogeneity within GBM [7, 13]. In recent years, there have been several published scRNA-seq datasets in gliomas [12, 14–19], which have helped elucidate the general differences between BMDM and microglial populations in the tumor microenvironment. However, each study has been limited in terms of patient numbers. Here, we combine 9 new scRNA-seq samples from glioblastomas with 57 previously published cases to explore the profiles of macrophages in gliomas at an unprecedented scale. In doing so, we identify a novel pro-tumor macrophage marker in GBM (MARCO) validated by immunofluorescence imaging. In addition to studying the clinical effects of MARCO expression on survival and during immunotherapy, we also characterize its associations with mesenchymal, hypoxic, and anti-inflammatory signatures.

## Methods

### Study design and data acquisition

Published single-cell RNA-seq count matrices were obtained from several repositories [20–25] and joined with 9 previously unpublished cases [26] to form a cohort of 50 GBM and 16 LGG samples (Additional file 1: Table S1). Bulk expression and survival data were obtained from 528 GBM cases from TCGA-GBM [27] and 75 GBM cases from Wang et al. [28]. Expression subtyping and IDH1 mutation status for TCGA were acquired from Ceccarelli et al. [29]. Gene sets were obtained from MSigDB v6.2 [30], with the exception of the BMDM versus microglia gene sets, which were obtained from Yuan et al. [17].

### Single-cell RNA-seq

The GBM specimens were collected from surgical resections of de-identified patients at Columbia University Irving Medical Center who provided informed consent to participate in these studies through a protocol approved by the Columbia Institutional Review Board (IRB-AAAJ6163). Two different methods were used to dissociate the tissue specimens into single-cell suspensions. PDC001, PJ052, PJ053, PW032-706All, and PW032-710All were dissociated using the method previously published by Yuan et al. [17]. PW039-705, PW035-710All, PW016-703_All, and PW017-703_All were dissociated into single cells using the Adult Brain Dissociation Kit (Miltenyi Biotec) on a gentleMACS Octo Dissociator with Heaters (Miltenyi Biotec) according to the manufacturer’s instructions. Single-cell suspensions were applied to an automated microwell-based platform for scRNA-seq library construction as previously described by Yuan et al. [17]. scRNA-seq libraries for PDC001, PW039-705, PW035-710All, PJ052, PJ053, PW016-703_All, and PW017-703_All were sequenced on an Illumina NextSeq 500 with an 8-base index read, a 21-base read 1 containing cell-identifying barcodes (CBs) and unique molecular identifiers (UMIs), and a 63-base read 2 containing the transcript sequence. The raw sequencing data were processed as described by Yuan et al. [17] to generate the digital gene expression matrices. scRNA-seq libraries for PW032-706All and PW032-710All were pooled and sequenced on an Illumina NovaSeq 6000 with an 8-base index read, a 26-base read 1 containing CBs and UMIs, and a 91-base read 2 containing the transcript sequence. The raw sequencing data were first corrected for index swapping to avoid cross-talk between sample index sequences [31] and then aligned to generate the digital gene expression matrices as described by Szabo et al. [32].

### scRNA-seq processing

All datasets were first filtered to remove mitochondrial and ribosomal proteins. Datasets were then merged together (separately for GBM and LGG) keeping the intersection of genes, with genes with zero total counts being discarded. Raw counts were then normalized to log_2_(1 + TPK), as described in Yuan et al. [17]. The intersection of expressed genes with LM22 from CIBERSORT [33] was used as a filtered list to reduce the batch effect. For visualization, first, principal components analysis (PCA) was applied to reduce the total dimensionality to 5% of the number of genes then uniform manifold approximation and projection (UMAP) [34] with default parameters to non-linearly reduce that into a two-dimensional embedding. Three cell types were readily identified using the standard markers of CD14, CD3, and SOX2 for macrophages, T lymphocytes, and tumor cells [17], respectively. The macrophages were isolated, and the same dimensionality reduction procedure described above was used to generate an embedding for the macrophage population. The embedding of the macrophage population was separated through *k*-means clustering. Silhouette scores were calculated from k=2 to k=6, with k=2 providing the highest silhouette score. This filtered list was used to assess the top enriched genes characterizing each of these two macrophage clusters.

### Immunofluorescence imaging

To validate the presence of MARCO in tissue from patients with GBM, we co-stained MARCO with CD163 using dual stain immunofluorescence (IF). Tissue specimens were evaluated from one patient with each tumor subtype, including IDH1-wild-type GBM, IDH1-mutant GBM, and grade III anaplastic astrocytoma (LGG). In addition, two patients treated with anti-PD1 inhibitors were compared pre- and post-anti-PD1, including a responder and a non-responder. IF staining was performed on 5-μm sections using anti-MARCO (1:100, PA5-64134, Invitrogen (Carlsbad, CA)) followed by biotinylated goat anti-rabbit IgG (1:200, BA1000, Vector Laboratories) and streptavidin-conjugated 594 (1:1000, S11127, Invitrogen), as well as anti-CD163 (1:100, 10D6, Biocare Medical (Pacheco, CA)) followed by biotinylated horse anti-mouse IgG (1:200, BA2000, Vector Laboratories) and streptavidin-conjugated 488 (1:300, Invitrogen). Stained sections were examined and photographed using a Nikon Eclipse TE2000-E. Composite images from emission wavelengths of 395, 488, and 568 were captured from each field of interest. All images in Fig. 4c were thresholded at a fixed value for the MARCO channel using ImageJ. For single-stain controls, all channels were thresholded at fixed values.

### Survival analysis

TCGA-GBM U133 microarray data and pre-treatment expression data from Wang et al. [28] were used to determine the expression of MARCO. Each cohort was first log- and Z-score normalized independently before being combined. These normalized values were merged with survival data, including overall and disease-free survival. Survival differences were assessed in two ways: (1) dichotomizing MARCO expression across the median into MARCO-high and MARCO-low then comparing the survival curves with the log-rank test and (2) generating a univariable Cox model directly based on the expression of MARCO and taking the Wald p-value of the MARCO covariate. In Fig. 2a and b, we only used cases that were known to be IDH1-wild-type.

### Expression subtype score

The single-cell subtype score method from Patel et al. [18] was used to calculate the signature score of each Verhaak expression type [35]. This same method was used to access the BMDM versus microglial signatures from Yuan et al. [17] as well as for assessing the combined score of the tumor-macrophage cross-talk genes.

### Gene set enrichment analysis

GSEA was performed with the official client v4.0.1 for Linux [30, 36]. The normalized gene expression of all macrophages was used as the input data, with MARCO expression as the phenotype label, and Pearson correlation as the metric for ranking genes. Default weighting (p = 1) was used. Phenotype permutation with 1000 iterations was used to calculate p-values, and the final gene sets were ranked by enrichment score.

### Tumor-macrophage cross-talk

The mean normalized expression of MARCO within the macrophage population was calculated for each GBM sample, and compared to a selected list of recruitment factors [37] within the tumor population of those same samples: macrophage colony-stimulating factor (CSF1), granulocyte/macrophage colony-stimulating factor (CSF2), hepatocyte growth factor (HGF), monocyte chemotactic protein 1 (MCP-1), macrophage inhibitory factor (MIF), stromal-derived factor 1 (SDF-1), transforming growth factor β (TGF-β, including TGFB1, 2, and 3), interleukin 10 (IL-10), osteopontin (SPP1), and lactadherin (MFGE8). An expression subtype score was calculated using this gene set and averaged across the tumor cells from each case. This score was then compared to MARCO expression in the corresponding macrophage population via Spearman correlation. The same procedure was used to assess the correlation of MARCO expression in macrophages with expression subtype scores in paired tumor cells.

### Dispersion analysis

To quantify the utility of MARCO as a marker compared to conventional myeloid, BMDM, and microglia markers, we assessed the normalized dispersion coefficient of each gene. We used the highly_variable_genes function in SCANPY [38] with default parameters and batch correction. We determined p-values with the exact permutation test.

### Statistical analysis

All statistical analyses were conducted in Python 3.6. In all boxplots, the center lines represent the median, lower and upper box limits are respectively the first and third quartiles, and whiskers represent the maximal values up to 1.5 times the interquartile range. All values extending beyond this range are considered fliers/outliers. Shaded regions in survival curves represent 95% confidence intervals. Violin plots use the Gaussian kernel to estimate densities, and the center lines represent the median. The two-sided Mann-Whitney U test was used throughout to non-parametrically compare two populations. p-values were adjusted for multiple comparisons using the Benjamini-Hochberg (FDR) procedure, and statistical significance was assessed at an adjusted p-value threshold of 0.05.

## Results

### Single-cell identification of a MARCO+ subpopulation of macrophages in GBM

**Fig. 1:**
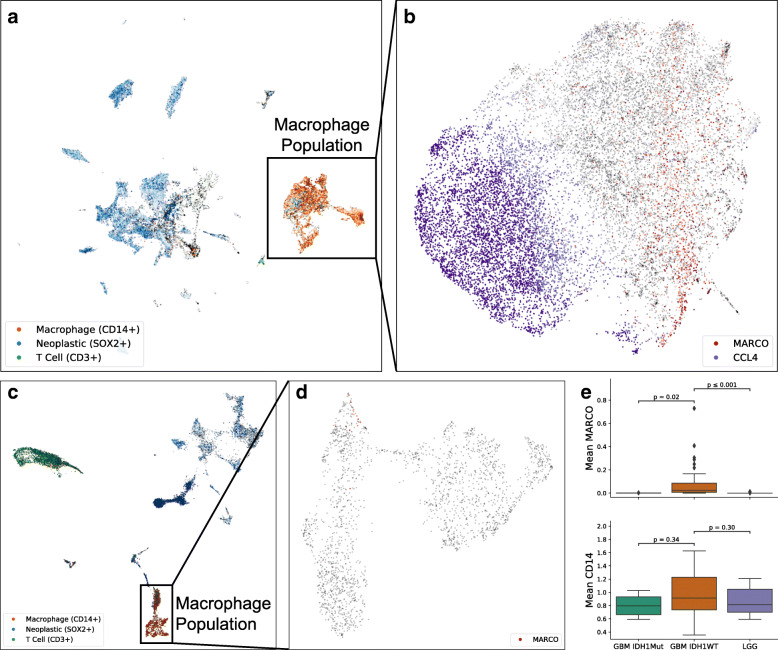
Identification via scRNA-seq of a MARCO+ subpopulation of macrophages specific to IDH1-WT glioblastoma. **a** Single-cell depiction of 79,968 glioblastoma cells reveals a macrophage population characterized by CD14. **b** Applying gene filtering and unsupervised clustering upon these macrophages reveals MARCO as defining an anti-inflammatory side opposite to CCL4. **c** Single-cell exploration of 18,047 lower-grade glioma cells reveals little MARCO expression within the macrophage population (**d**). **e** The mean MARCO expression among macrophages is specific to IDH1-wild-type GBM (above), while the mean CD14 expression among macrophages is similar (below)

To understand the heterogeneity of cellular profiles in GBM, we collected single-cell RNA-seq data from nine GBM cases and combined it with 41 GBM cases from previously published studies for a total of 79,968 single-cell transcriptomes (Additional file 1: Table S1). To reduce the batch effect, we filtered our gene list to the 499 genes that overlapped with LM22, the reference matrix used by CIBERSORT [33] to differentiate immune cells (Additional file 2: Table S2). After filtering, log-normalization, batch effect reduction, and dimensionality reduction, visualization of these cells produced a distinct macrophage population of 17,132 cells characterized by CD14 (Fig. 1a, Additional file 3: Fig. S1). Unsupervised *k*-means clustering on this macrophage population revealed two subpopulations (validated by silhouette score, Additional file 3: Fig. S2). One of these subpopulations was enriched in inflammation-related genes, with CCL4 and IL1B as the top two genes on the filtered gene list (p = 0.004 and p = 0.008, respectively, exact permutation test, n = 499 genes; Fig. 1b). The other subpopulation, opposite to the inflammatory side, had MARCO (macrophage receptor with collagenous structure) as the top gene (p = 0.004, exact permutation test, n = 499 genes; Fig. 1b). MARCO remained the top enriched gene for this side even when left out during the dimensionality reduction, demonstrating its representativeness (p = 0.004, exact permutation test, n = 499 genes). Looking at the expression of MARCO in all cell populations, we found that it is specifically found in this subpopulation of macrophages (Additional file 3: Fig. S3A). MARCO is a scavenger receptor normally found on alveolar macrophages [39] with a variety of immunomodulatory roles [40–42]. Given the negative role of MARCO in other cancers [43–45], we chose to focus on these MARCO+ macrophages and their impact on the tumor microenvironment.

### Absence of MARCO expression in LGG and IDH1-mutant GBM

We next investigated whether this macrophage subpopulation could also be observed in lower-grade gliomas (LGGs), which include grade II and III astrocytomas as well as oligodendrogliomas. We collected single-cell expression profiles of 18,047 cells from 16 LGGs and processed them in the same manner as for GBM above (Fig. 1c). Although 2199 macrophages were identified, only 1% (23 cells) had non-zero expression of MARCO (Fig. 1d) compared to the 12% in GBM (2092 cells out of 17,132, *p* < 0.001; chi-squared test). This is despite the GBM samples having lower library complexity (*p* < 0.001, n = 49 patients, Mann-Whitney U test; Additional file 3: Fig. S4). Since most LGGs are IDH1-mutated, we examined MARCO expression in the 4 GBM cases with IDH1 mutations. Of the 281 macrophages from IDH1-mutated GBMs, only one cell had non-zero expression of MARCO. Comparing normalized expression levels, the mean MARCO expression in IDH1-wild-type GBM macrophages was 160 times higher than in IDH1-mutated GBM (p = 0.020, n = 41 patients, Mann-Whitney U test) and 64 times higher than in LGGs (p = 0.0007, Fig. 1e top, n = 49 patients). Meanwhile, the mean expression of other macrophage markers such as CD14 was not significantly different (p = 0.34 and p = 0.30, Fig. 1e bottom, n = 41 and n = 49 patients). Immunofluorescence imaging in IDH1-wild-type cases validates that MARCO co-localizes with CD163 macrophages in tissues from these patients, while IDH1-mutated and LGG tissues show rare, if any, co-localization (Additional file 3: Figs. S5 and S6). Therefore, MARCO expression is almost exclusively found in macrophages of IDH1-wild-type GBM, rather than their less deadly IDH1-mutated or lower-grade counterparts.

### MARCO bulk expression associates with poor clinical outcomes and mesenchymal subtype

**Fig. 2:**
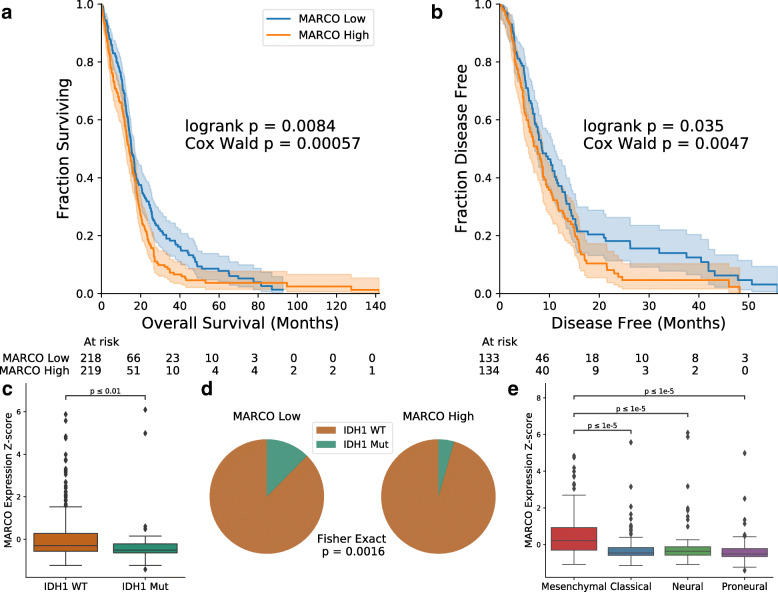
Bulk MARCO expression in GBM is associated with poor clinical prognosis and mesenchymal subtype. MARCO expression in TCGA-GBM is associated with poorer overall survival (**a**) and disease-free survival (**b**). **c**, **d** Bulk MARCO expression also associates with IDH1-wild-type status. **e** TCGA-GBM expression subtyping shows an enrichment of MARCO with the mesenchymal subtype

To understand the clinical consequences of MARCO expression, we examined its bulk expression in two published datasets: TCGA-GBM and the GBM cohort from Wang et al. [28] (for a total of n = 603 patients). Splitting patients into the MARCO-high and MARCO-low groups down the median, we found a negative association of MARCO expression with both overall survival (OS: p = 0.0046, n = 592 patients, log-rank test, Additional file 3: Fig. S7A) and disease-free survival (DFS: p = 0.018, n = 387 patients, Additional file 3: Fig. S7B). However, given the aforementioned association of MARCO with IDH1 and the strong impact of IDH1 mutations on survival, we repeated the analysis with only the 437 GBMs with known IDH1-wild-type status, and the effect persisted (OS: p = 0.0084, n = 437 patients; DFS: p = 0.035, n = 267 patients, log-rank test, Fig. 2a, b). Continuing this analysis with the IDH1-wild-type cohort, we treated MARCO expression as a continuous variable and found that the effect of MARCO in a univariable Cox model was also significantly detrimental to survival (p = 0.00057 for OS, n = 437 patients, hazard ratio = 1.19; p = 0.0047 for DFS, n = 267 patients, hazard ratio 1.19; Wald test). These effects were most pronounced in terms of long-term survival, with MARCO-high patients having roughly half the overall survival rate at the 2-year (18.0% vs. 31.8%) and 5-year (3.7% vs. 7.8%) time points compared to MARCO-low patients. Similarly, 2-year DFS was over three times lower in the MARCO-high population (5.8% vs. 18.1%; no data for 5-year DFS). The effect of MARCO expression on survival remained significant after controlling for common confounders including age and MGMT methylation status (Additional file 3: Fig. S7C).

Recapitulating the single-cell results, we found an association of IDH1 mutations with decreased MARCO expression in bulk data (p = 0.0039, n = 482 patients, Mann-Whitney U test, Fig. 2c). This was also significant with dichotomizing the population into MARCO-high and MARCO-low patients (p = 0.0016, n = 482 patients, Fisher Exact test, Fig. 2d). We then compared MARCO bulk expression with transcriptomic subtype [29]. Although MARCO is not on the original list of mesenchymal genes [35], its expression was highly enriched in mesenchymal samples (*p* < 0.00001 for all pairwise comparisons between mesenchymal and other subtypes, n = 565 patients; Fig. 2e). These bulk transcriptomic results support our single-cell findings of the enrichment of MARCO in IDH1-wild-type tumors and also demonstrate an association of MARCO expression with worse prognosis and the unfavorable mesenchymal subtype.

### Single-cell association of MARCO with mesenchymal traits and hypoxia

**Fig. 3:**
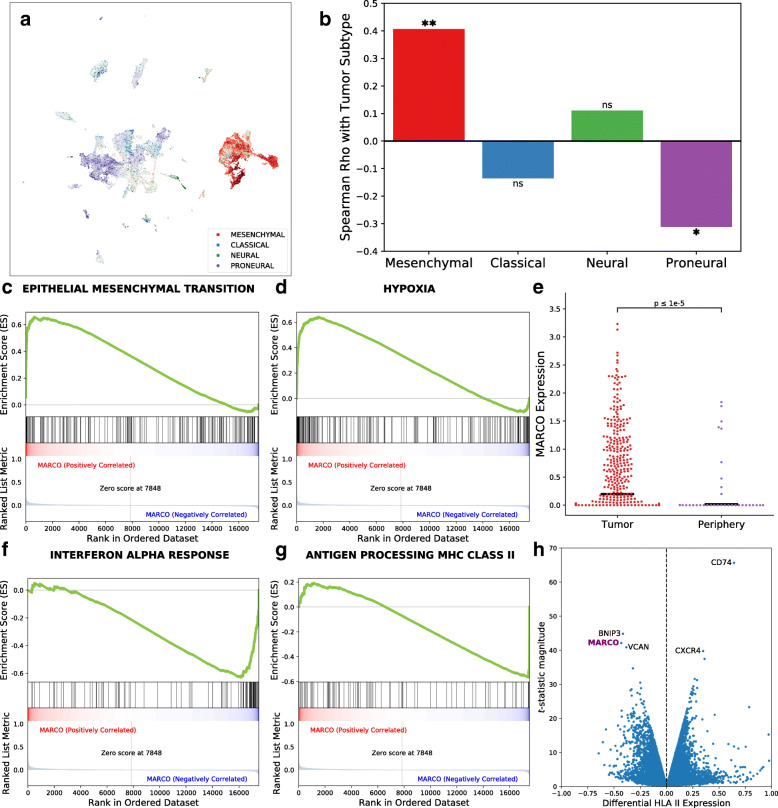
Single-cell characterization of the pro-tumor features of MARCO+ macrophages. **a** The mesenchymal signature primarily originates from the macrophage population. **b** Single-cell expression analysis from paired macrophages and tumor cells from the same samples. The mean MARCO expression within macrophages significantly correlates with mesenchymal subtype score and anti-correlates with proneural subtype score in paired tumor cells. *: p < 0.05; **: p < 0.01; ns: not significant. **c** MARCO+ macrophages demonstrate an enrichment in gene sets related to the epithelial-mesenchymal transition. **d** The hypoxia pathway is also enriched in MARCO+ macrophages. **e** This corroborates with their presence in the tumor core rather than the tumor periphery in 4 pairs of locationally separated samples in Darmanis et al. (black lines in the swarm plots represent means). **f** Pathways least expressed by MARCO+ macrophages include interferon-alpha response as well as MHC II antigen presentation (**g**). **h** Out of all genes surveyed, MARCO expression is distinctly associated with decreased HLA class II gene expression

To uncover the source of this mesenchymal signature, we went back to our single-cell expression data. The mesenchymal expression signature was found primarily in the macrophage population rather than in tumor cells (*p* < 0.00001, n = 50 patients, Mann-Whitney U test, Fig. 3a, Additional file 3: Fig. S8, Additional file 2: Table S3) and specifically within MARCO-expressing macrophages (p = 0.005). MARCO mean expression in macrophage cells also significantly correlated with the mesenchymal signature score in tumor cells from the same sample (p = 0.0084, n = 41 patients, Spearman correlation; see the “Methods” section; Fig. 3b), while anticorrelating with the proneural signature (p = 0.047). We then performed gene set enrichment analysis (GSEA) based on the Pearson correlation of all other genes with MARCO expression to understand what processes underlie this subpopulation. Out of the 50 hallmark gene sets from MSigDB, the gene sets with the highest enrichment scores among MARCO+ macrophages were epithelial-mesenchymal transition, angiogenesis, glycolysis, and hypoxia (FDR q < 0.001 for all four, permutation test on phenotypes with default weighting, n = 17,132 cells; Fig. 3c, d). This association with hypoxia is supported by single-cell data from Darmanis et al. [14], in which samples were taken from the core and periphery of the same four tumors. Macrophages from the tumor core had higher MARCO expression than those taken from the tumor periphery (*p* < 0.001, n = 1846 cells; Mann-Whitney U test; Fig. 3e). Furthermore, we investigated the expression of MARCO in Ivy GAP [46], a database with laser-microdissected specimens from different anatomic structures. We found significantly different expression of MARCO in various structures (p = 0.014, n = 270 specimens from 37 patients; Kruskal-Wallis test, Additional file 3: Fig. S9), with the highest expression in the perinecrotic zone within the cellular tumor. These spatially informed findings support the GSEA characterization of MARCO+ macrophages as residing in the hypoxic tumor core.

### MARCO+ macrophages demonstrate loss of inflammatory pathways and antigen presentation

Since hypoxia can polarize macrophages toward a pro-tumor phenotype [47], we also investigated if appropriate antitumoral responses were downregulated within MARCO+ macrophages. Also via GSEA, we found that the gene sets with the lowest enrichment scores among the hallmark set were all pro-inflammatory: interferon alpha response, interferon gamma response, allograft rejection, and TNFa signaling via NFKB (FDR q ≤ 0.001 for all four sets, n = 17,132 cells, Fig. 3f). Interestingly, among the eight individual genes with the lowest relative expression in MARCO+ macrophages, four of them were HLA class II genes (HLA-DRB1, DRA, DPA1, and DPB1), and another was CD74 (MHC class II invariant chain). Accordingly, the GO gene set antigen processing and presentation of peptide or polysaccharide antigen via MHC class II was highly downregulated in MARCO+ macrophages (*p* < 0.001, n = 17,132 cells, Fig. 3g). Similarly, by comparing the differential expression of HLA class II genes in relation to all 17,496 other assayed genes, MARCO was one of the three most negatively associated (Fig. 3h) alongside BNIP3 (an apoptotic Bcl-2 family gene) and VCAN (an extracellular protein implicated in metastasis [48]). Loss of HLA class II expression in macrophages is generally associated with an anti-inflammatory, inactivated state [49] and has been previously linked to worse outcomes in melanoma [50] as well as unfavorable tumor profiles in the context of PD1 immunotherapy in GBM [7].

### Dynamics of MARCO expression under PD1 immunotherapy

**Fig. 4:**
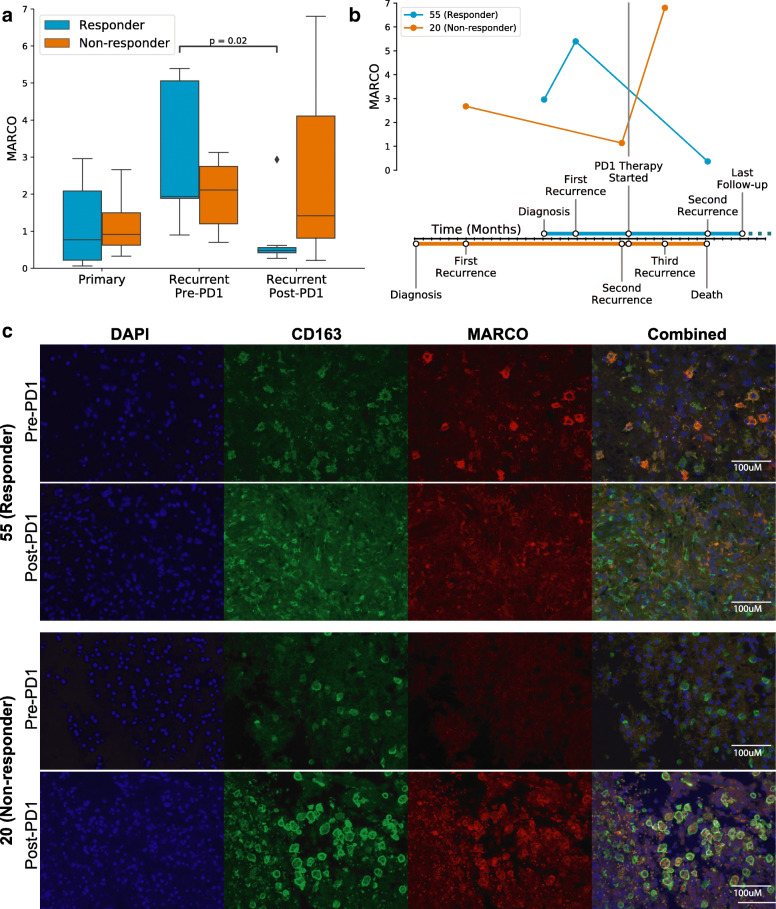
Dynamics of MARCO expression under PD1 immunotherapy. **a** Comparison of bulk MARCO expression in a longitudinal cohort of GBM patients treated with adjuvant PD1 checkpoint inhibitors following recurrence. Immunotherapy responders showed a decrease in MARCO expression between pre- and post-immunotherapy recurrences. **b** Timeline of representative examples of a responder (patient 55, blue) and non-responder (patient 20, orange) to PD1 immunotherapy. MARCO expression levels from available samples are plotted concurrently with the disease course. Each tick on the *x*-axis represents 1 month of time. **c** Immunofluorescence imaging of these same two representative cases (patients 55 and 20) before and after anti-PD-1 therapy, with dual staining of CD163 (green) and MARCO (red), alongside DAPI (blue)

To determine if the anti-inflammatory properties of MARCO+ macrophages play a role in PD1 checkpoint inhibitor therapies, we investigated MARCO expression in a longitudinal cohort of 17 PD1-treated GBM patients [7]. Interestingly, there was a decrease in MARCO between pre- and post- immunotherapy recurrences (p = 0.02, n = 37 time points; Mann-Whitney U test; Additional file 3: Fig. S10A), but this was solely found within responders (p = 0.02, n = 19 time points from 10 patients; Fig. 4a). A timeline of two representative cases from this cohort shows a responder that had a strong decrease in MARCO expression following immunotherapy, whereas a non-responder had an increase in MARCO expression after immunotherapy, followed soon after by death (Fig. 4b). Immunofluorescence imaging of tissues from these same two cases visually recapitulates these bulk transcriptomic findings (Fig. 4c, Additional file 3: Fig. S11). While this supports the negative role of MARCO in the long-term following adjuvant therapy, the dynamics may differ in the short-term—in an orthogonal study of neoadjuvant PD1 therapy in GBM, MARCO was reported as one of the top increased genes in patients treated with pembrolizumab approximately 2 weeks prior to sample acquisition [51]. Meanwhile, within a longitudinal cohort of 86 patients treated with standard therapy [28], we found no significant difference in MARCO expression before and after treatment (p = 0.21, n = 160 time points, Additional file 3: Fig. S10B), suggesting that these changes in MARCO are specific to immunotherapy.

### Recruitment of MARCO+ macrophages from the blood by tumor cells

**Fig. 5:**
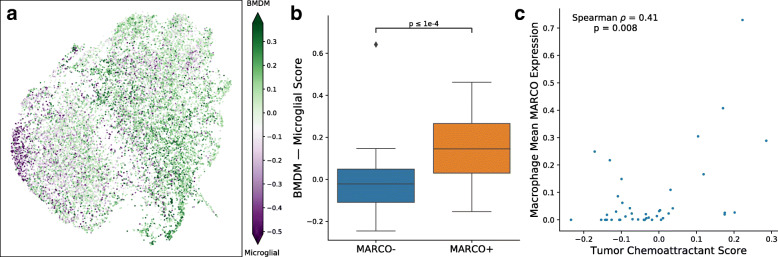
MARCO+ macrophages are recruited from the blood likely via tumor signaling pathways. **a** Assessment of BMDM versus microglia gene sets reveals that the MARCO+ macrophage population is more similar to BMDMs (**b**). **c** Single-cell expression from paired macrophage-tumor cells from the same samples reveals that the mean MARCO expression within macrophages correlates with the expression score of a chemoattraction signature in tumors

One important question for the potential targeting of MARCO+ macrophages is where they originate from and how they are recruited to the tumor. Based on the gene sets associated with BMDMs versus resident microglia [17], we found that MARCO+ macrophages more closely resembled BMDMs (*p* < 0.0001, n = 71 subpopulations; Mann-Whitney U test; Fig. 5a, b). Compared to non-MARCO-expressing macrophages, CD163 was among the highest differentially expressed genes in MARCO+ macrophages (p = 0.0037, n = 17,509 genes; exact permutation test), and TMEM119 was among the lowest (p = 0.0055), which are classic markers characterizing BMDMs and microglia, respectively. Nonetheless, MARCO appears to define a more specific subpopulation than solely BMDM-microglial differences (p = 0.004, n = 17,509 genes; see the “Methods” section; Additional file 3: Fig. S3B).

To understand the factors that recruited these macrophages, we examined the expression of a list of 10 genes known to attract and re-program macrophages in GBM [37]. We found a significant positive correlation of MARCO mean expression in macrophage cells with the corresponding normalized expression of these recruitment factors in tumor cells from the same sample (p = 0.008, n = 41 patients, Spearman correlation; see the “Methods” section; Fig. 5c). These results are supported by correlations in bulk expression data from TCGA-GBM, in which MARCO is positively associated with this same signature of recruitment factors (*p* < 0.001, n = 528 patients, Spearman correlation; see the “Methods” section; Additional file 3: Fig. S12). Furthermore, we found within our GBM single-cell data that there is significant co-expression of Ki-67 with MARCO (*p* < 0.00001, n = 17,132 cells, chi-squared test; Additional file 3: Fig. S13), suggesting that these MARCO+ macrophages may also be proliferating within the tumor microenvironment.

## Discussion

In this manuscript, we have characterized the role of MARCO as a marker of pro-tumor macrophages in GBM. In particular, we have found that this MARCO-expressing subpopulation associates with mesenchymal, hypoxic, and anti-inflammatory traits as well as poor clinical prognosis. The mesenchymal nature of MARCO is supported by the role of MARCO in regulating the epithelial-mesenchymal transition outside the context of cancer [41]. Meanwhile, the upregulation of glycolysis and hypoxia gene sets is consistent with the enrichment of MARCO+ macrophages in the tumor core—which is known to be associated with pro-tumor macrophages [52]. Furthermore, the localization of these macrophages within the hypoxic, necrotic core is consistent with the observations that MARCO is upregulated in regions of ischemic brain, suggesting a functional role of this scavenger receptor in clearing cellular debris [53]. Our finding of the opposition of MARCO to inflammatory genes and pathways is also seen in mice, where tolerized BMDMs upregulate MARCO [40]. In fact, the remarkable downregulation of HLA class II genes on MARCO+ macrophages is consistent with murine studies where MARCO expression is associated with a decrease of antigen internalization capacity [42]. Although MARCO has been previously reported as a pro-tumor TAM marker in non-small cell lung cancer [43], lung adenocarcinoma [54], and breast cancer [44], and has been associated with poor prognosis in periampullary adenocarcinoma [45], its role has not been previously reported in GBM. Our findings of the pro-tumor TAM traits associated with MARCO in GBM adds credence to its importance across cancers.

Here, we show that MARCO+ macrophages appear to be recruited from the blood via the upregulation of a set of factors secreted by tumor cells, including CSF1 and TGF-β. CSF1 expression in tumor cells has been previously shown to be related to higher proportions of TAMs in GBM [17]. Notably, these TAMs were observed to express the cognate receptor CSF1R, which is targetable by existing therapeutics [11, 17]. Meanwhile, TGF-β has been experimentally shown to upregulate MARCO expression in M0 BMDMs [44], joining a host of other studies implicating TGF-β in glioma progression [28, 55]. While these recruitment factors are potential targets, MARCO itself also presents a promising target—anti-MARCO therapeutic antibodies have demonstrated efficacy in mouse melanoma models [44, 56]. As with other immunotherapies, we do not expect monotherapy with anti-MARCO antibodies to markedly improve outcomes alone. However, including anti-MARCO in combination regimens may be advantageous. While MARCO expression remains unaltered across standard therapy, we found that its expression changes in the course of anti-PD1 immunotherapy, with responders exhibiting decreases in MARCO in the long term following treatment. This result suggests that MARCO+ macrophages may be detrimental to checkpoint immunotherapy, which has been largely unsuccessful in GBM [57]. Simultaneous targeting of MARCO or its associated recruitment factors may provide a promising opportunity to manipulate macrophage polarization and thereby boost the efficacy of checkpoint inhibitor therapy [58, 59]. Furthermore, there is a critical need for markers of response to anti-PD1 therapies. Previously, we had shown that mutations in MAPK genes and loss of PTEN predict response to anti-PD1 immunotherapy [7]. However, there is no current marker of response after treatment with anti-PD1 inhibitors. MARCO may be useful to satisfy this need, and validation of this in prospective trials with anti-PD1 therapy could be beneficial.

## Conclusions

In this work, we utilized single-cell RNA sequencing to identify a pro-tumor subpopulation of macrophages characterized by MARCO expression. These macrophages are associated with both worse clinical outcomes as well as with the mesenchymal subtype. Furthermore, these macrophages appear to be derived from the bone marrow and affected by anti-PD1 checkpoint inhibitors. These results highlight a novel macrophage subpopulation that contributes to tumor progression in glioblastomas and suggest potential therapeutic strategies for its mitigation.

## Supplementary Information

**Additional file 1: Supplementary table S1.**

**Additional file 2: Supplementary tables S2 and S3.**

**Additional file 3: Supplementary figures.**

**Additional file 4.** **Source data for all figures.**

## Data Availability

All novel sequencing data have been deposited in the Gene Expression Omnibus under accession number GSE141383: https://www.ncbi.nlm.nih.gov/geo/query/acc.cgi?acc=GSE141383 [26]. All previously published datasets can be found in their respective public repositories [20–25]. The processed and source data underlying each figure are provided in Additional file 4. All custom code is available at https://github.com/RabadanLab/MARCOsinglecell [60].

## Abbreviations

BMDM: Bone marrow-derived macrophage
CB: Cell-identifying barcode
DFS: Disease-free survival
GBM: Glioblastoma
GSEA: Gene set enrichment analysis
IF: Immunofluorescence
LGG: Lower-grade glioma
OS: Overall survival
scRNA-seq: Single-cell RNA sequencing
TAM: Tumor-associated macrophage
UMAP: Uniform manifold approximation and projection
UMI: Unique molecular identifier
