# Supplementary material for: Single-cell characterization of macrophages in glioblastoma reveals MARCO as a mesenchymal pro-tumor marker
Source: Genome Med. 2021 May 19;13:88. doi: 10.1186/s13073-021-00906-x (PMC8136167; doi:10.1186/s13073-021-00906-x)
Supplement: Supplementary file 3 — Additional file 3: Supplementary figures. [file 13073_2021_906_MOESM3_ESM.pdf]

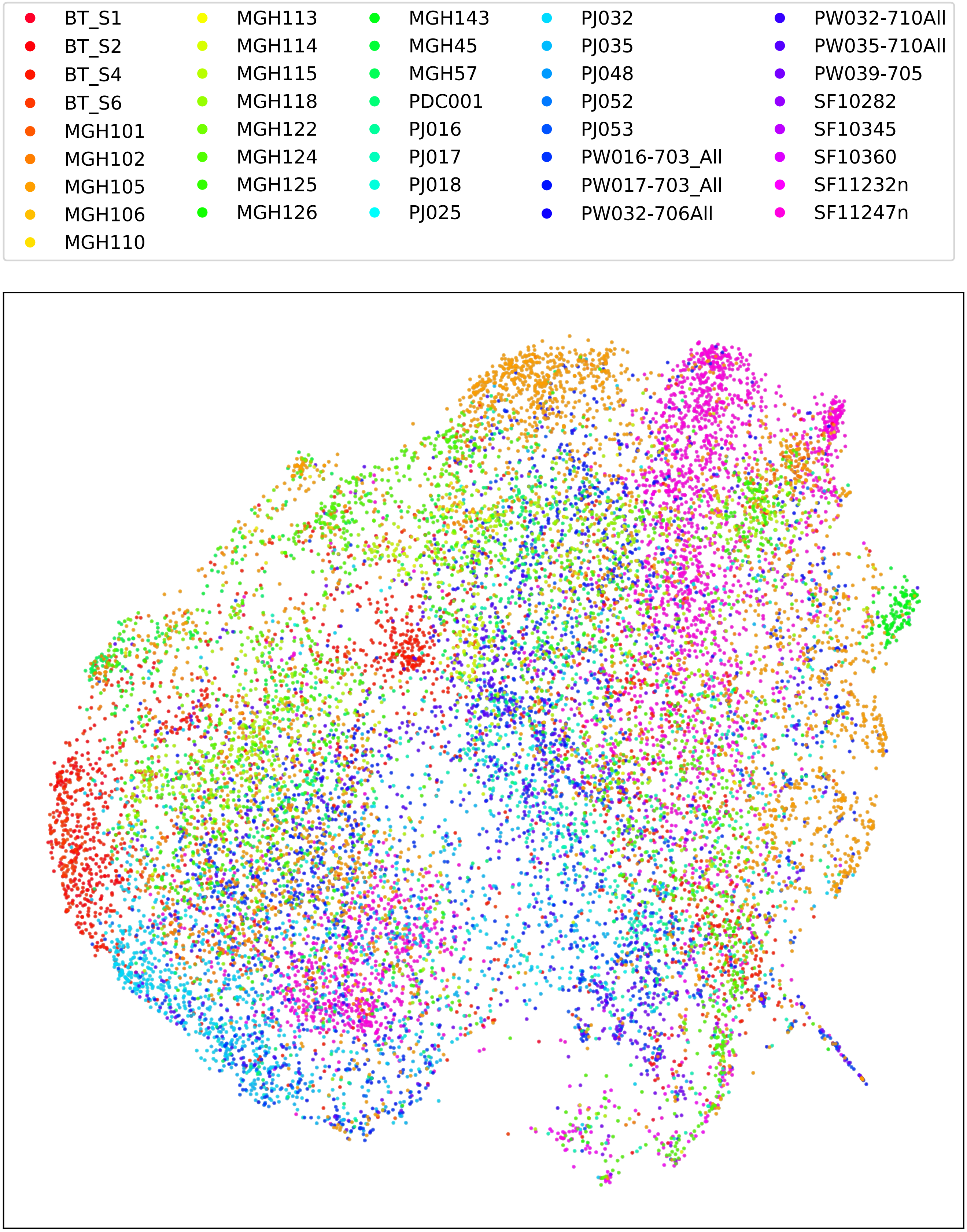

Fig. S1: Using a filtered subset of immune genes from CIBERSORT decreases batch effect in macrophages. UMAP embedding of the macrophage population within GBM (Fig. 1B), labeled by batch.

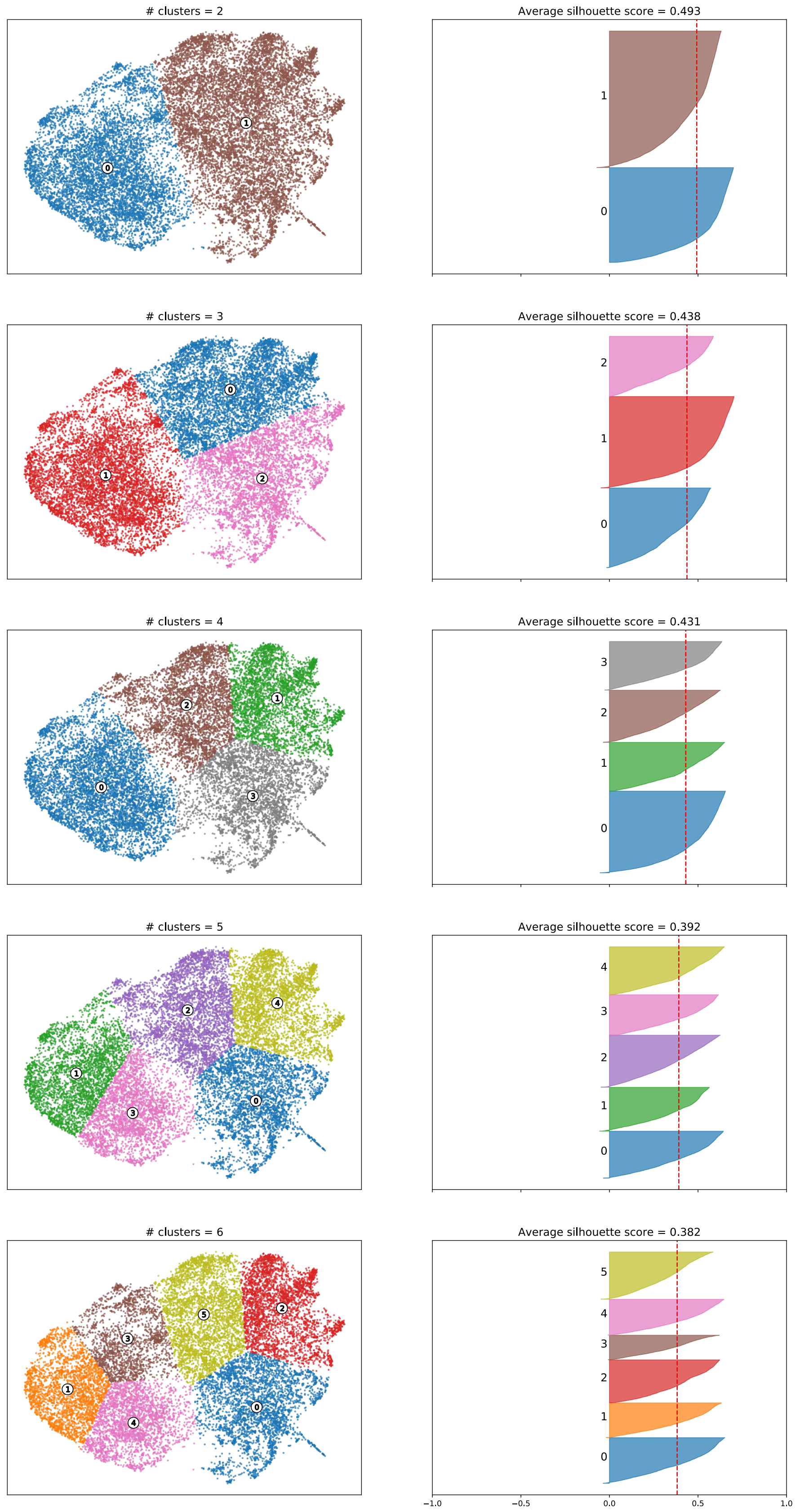

Fig. S2: Silhouette score justification for k = 2 clustering. k-means clustering of the macrophage population within GBM, varying from k = 2 to k = 6 clusters. The silhouette score is highest at k = 2.

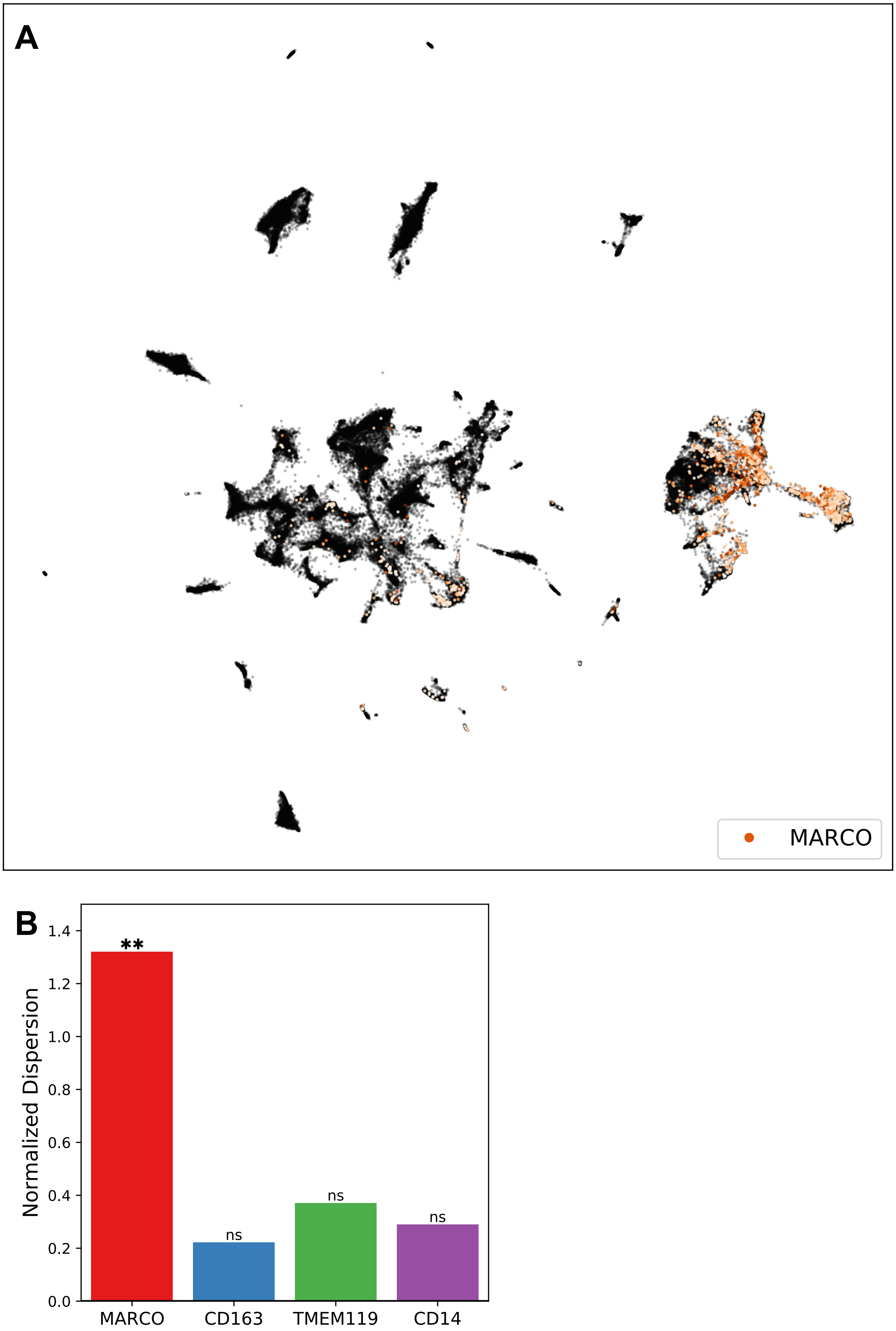

Fig. S3: MARCO is a macrophage-specific subpopulation marker. (A) Expression of MARCO across all GBM cells shows concentration in the macrophage compartment. Same embedding as Fig. 1A. **(B)** Normalized dispersion values for MARCO within the macrophage subpopulation compared to other macrophage markers. Higher dispersion indicates that a gene exists in a more specific subpopulation. *: $p < 0.05$; **: $p < 0.01$; ns: not significant.

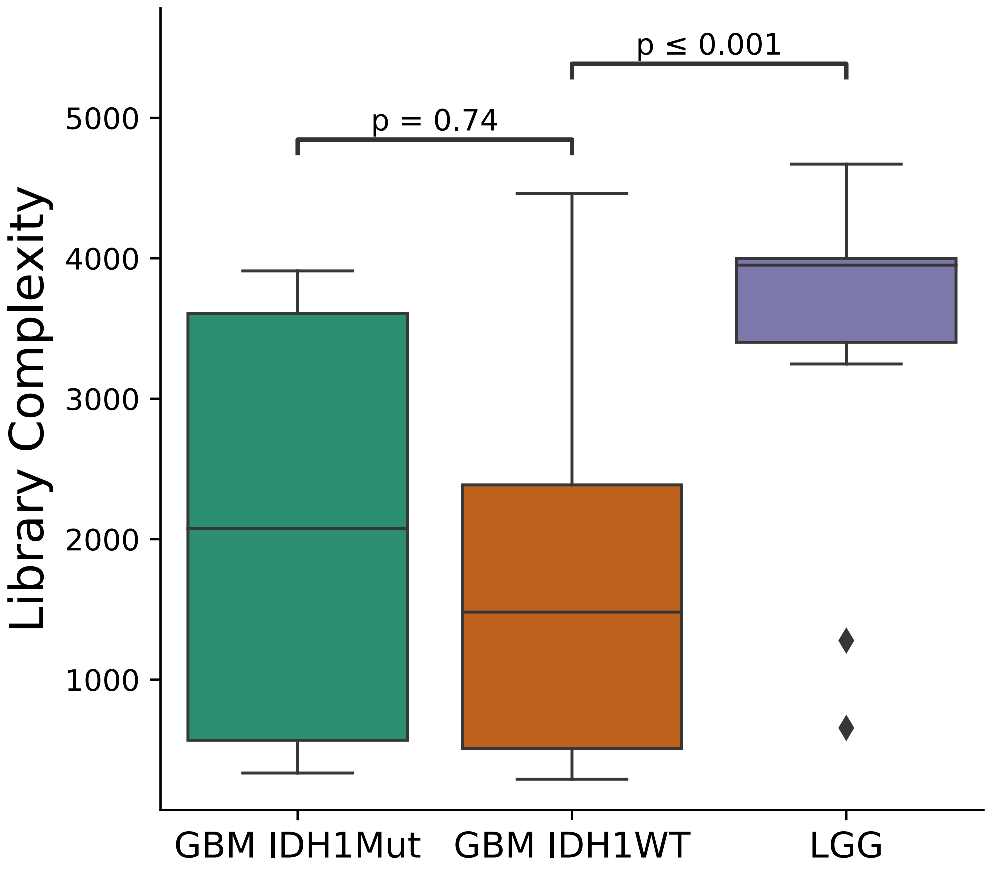

Fig. S4: Sample library complexity. IDH1-wildtype GBMs have lower library complexities averaged across each sample than their LGG counterparts.

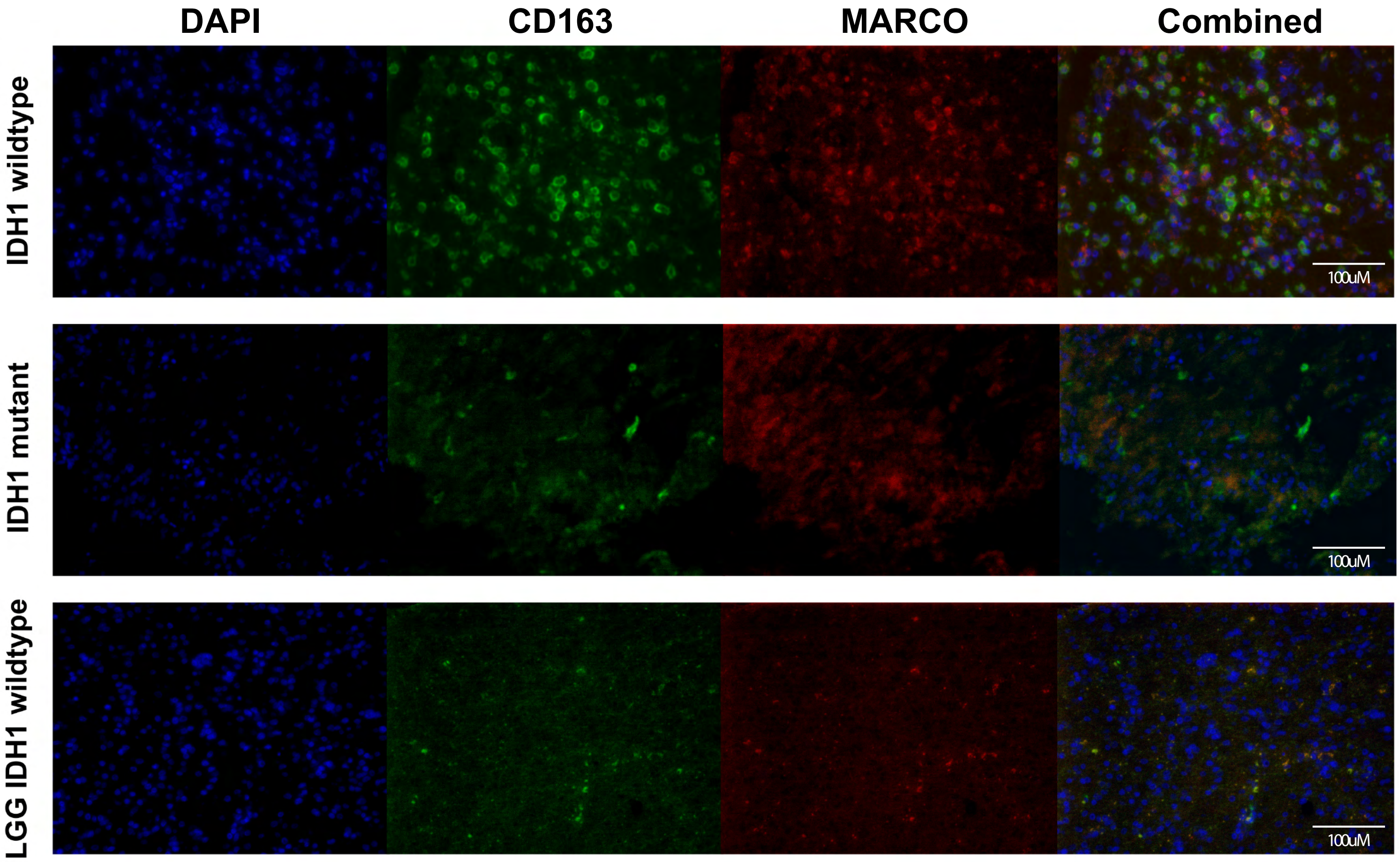

Fig. S5: Immunofluorescence imaging comparing MARCO expression in IDH1 wild-type, IDH1-mutated and LGG cases. Images show dual staining of CD163 (green) and MARCO (red), alongside DAPI (blue).

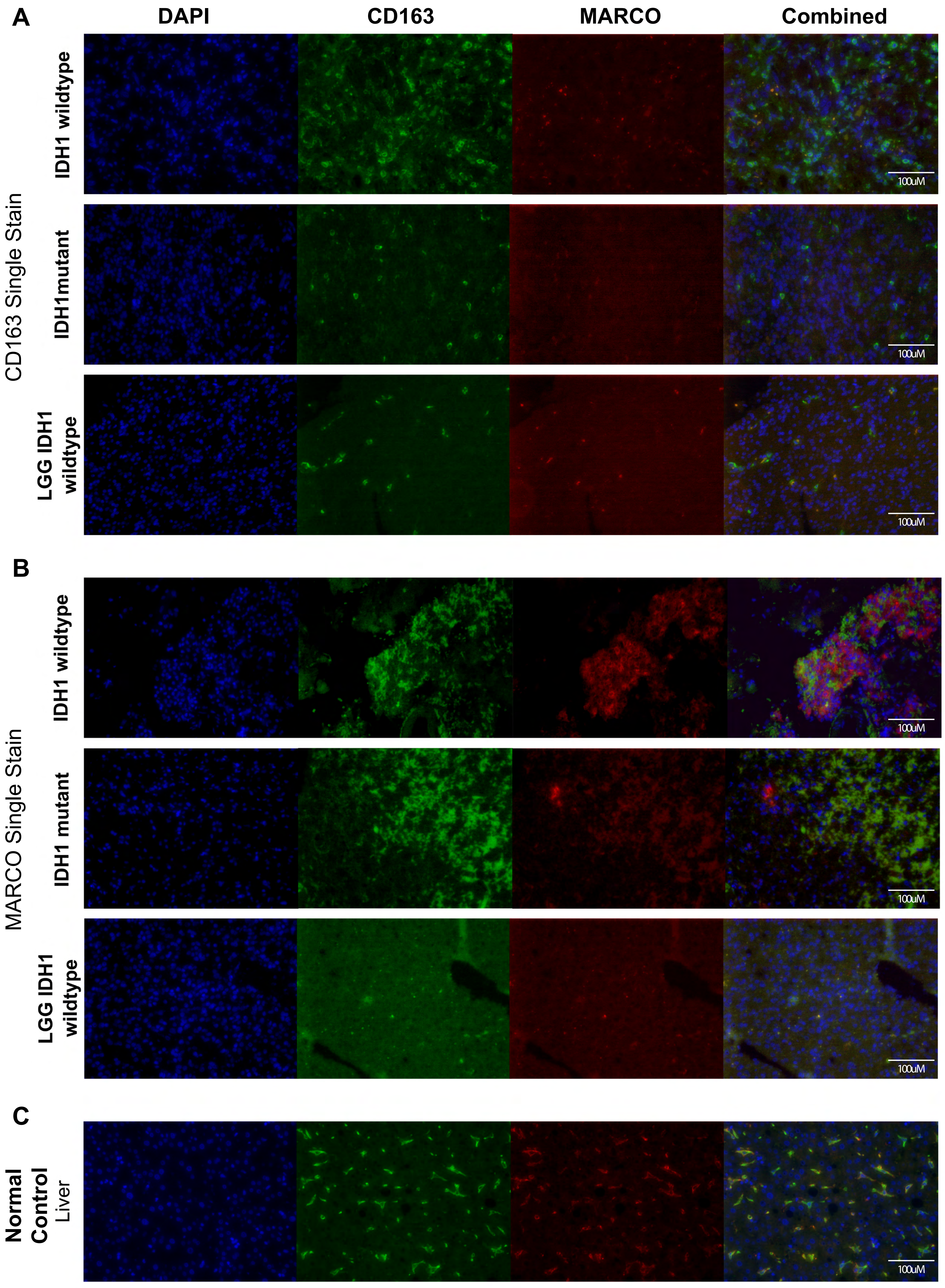

Fig. S6: Immunofluorescence imaging of MARCO+ macrophages across subtypes. Single-stain controls of **(A)** CD163 and **(B)** MARCO in the same samples as Additional file 3: Fig. S5. **(C)** Dual staining of normal liver control with the same markers.

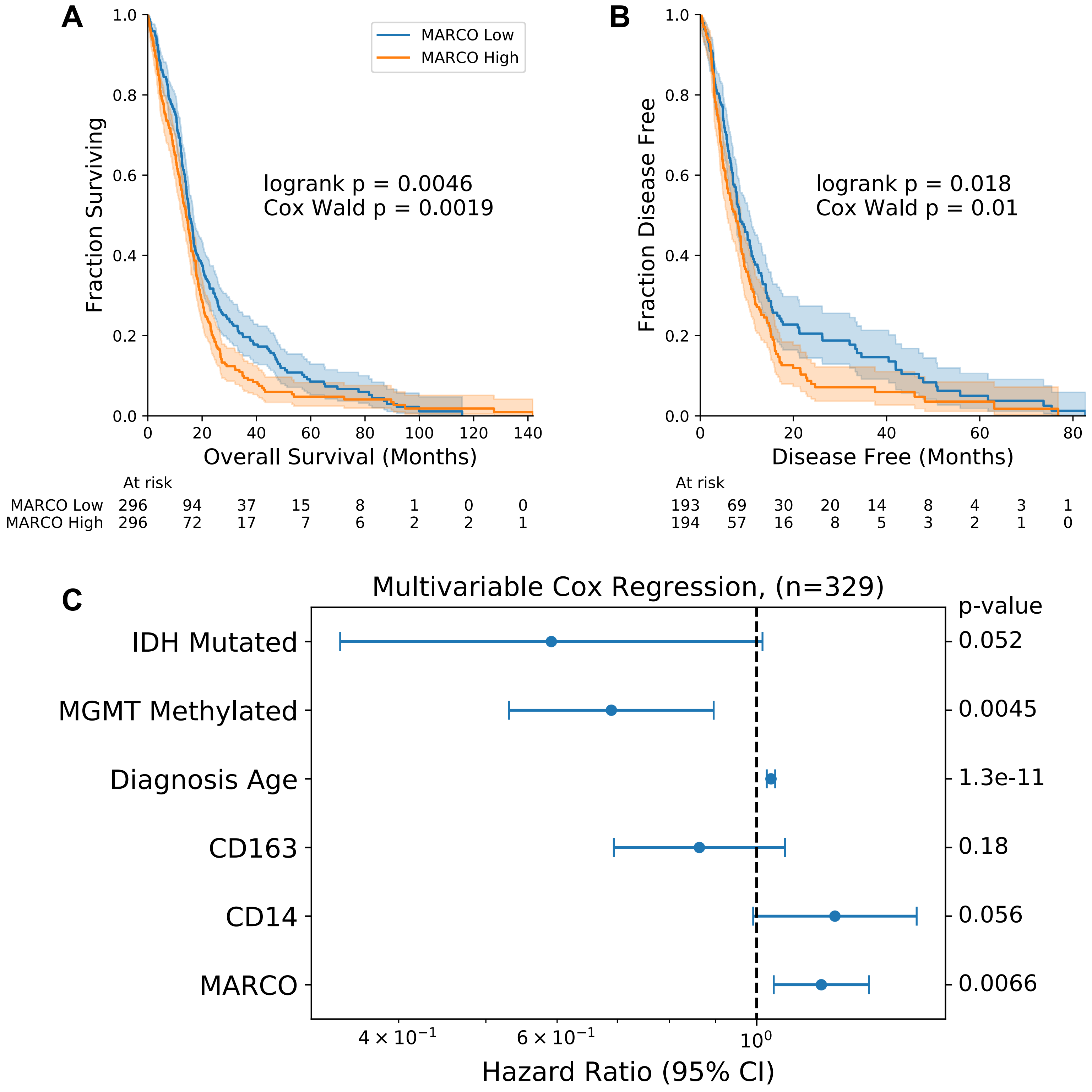

Fig. S7: MARCO bulk expression associates with poor survival. Survival analysis of overall **(A)** and disease-free survival **(B)** in public GBM datasets using the same method as Figs. 2A, B, but including GBMs of all IDH1 mutation statuses. **(C)** Multivariable Cox regression utilizing all 329 cases with complete information IDH and MGMT status as well as transcriptomic data. CD14 and CD163 serve as control markers for macrophages. Error bars represent 95% confidence intervals.

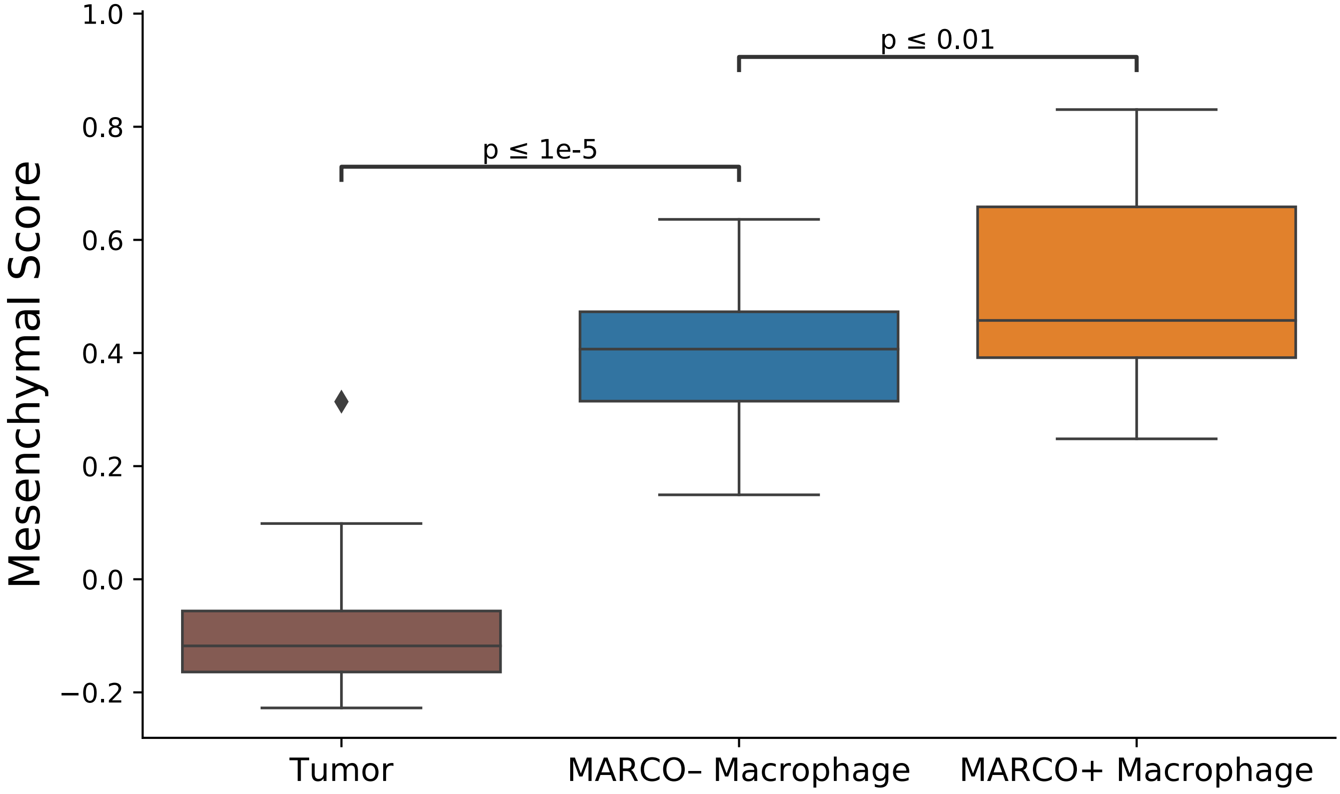

Fig. S8: Mesenchymal score is specifically enriched in MARCO+ macrophages. Quantification of Mesenchymal signature score from Fig. 3A. Macrophages have higher scores than tumor cells, and MARCO+ macrophages are still higher than MARCO– macrophages.

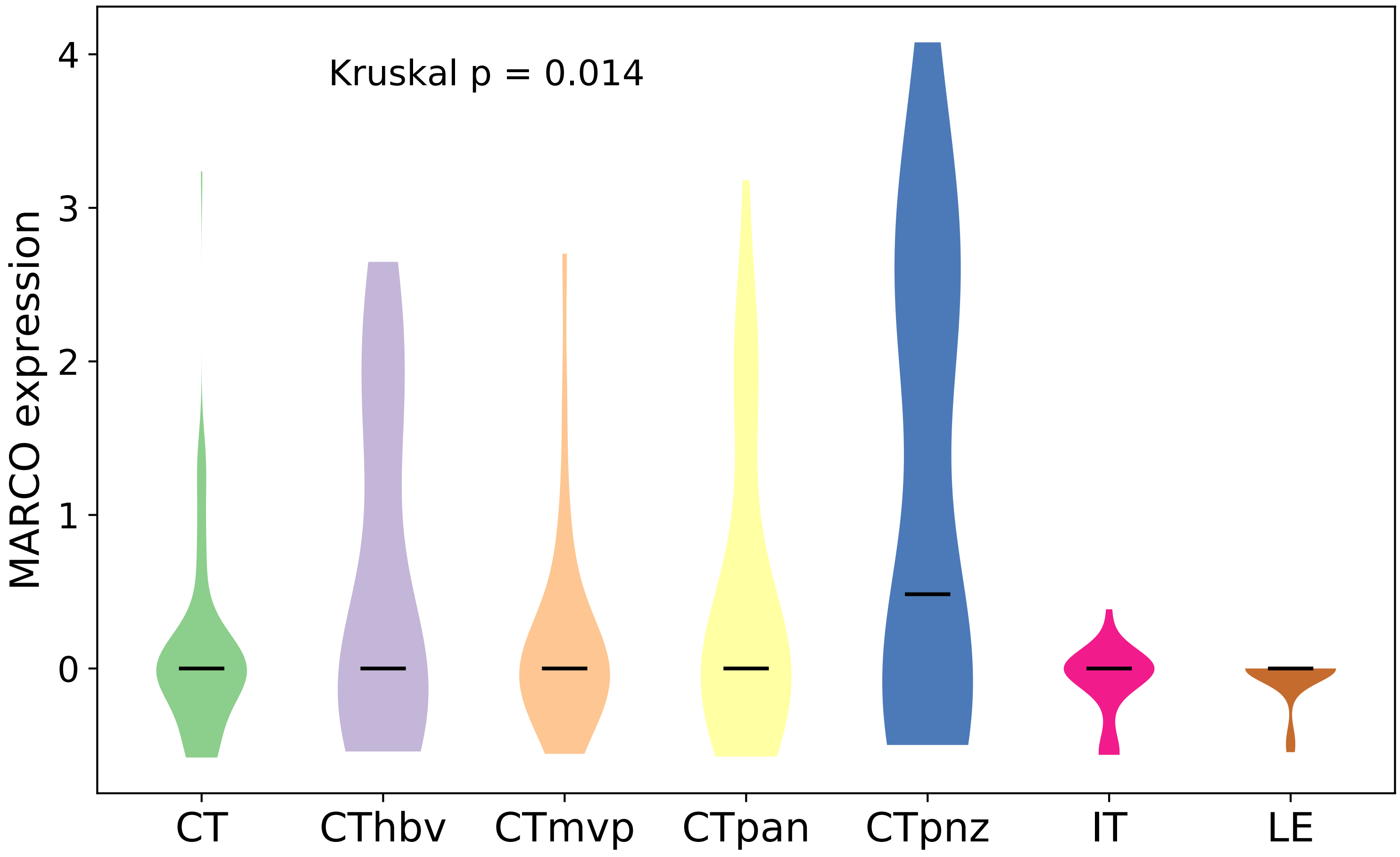

Fig. S9: MARCO expression in different anatomic structures. Relative expression (z-scores) of MARCO in different structures in GBM as separated by laser microdissection. CT = Cellular Tumor; hbv = Hyperplastic blood vessels; mvp = Microvascular proliferation; pan = Pseudopalisading cells around necrosis; pnz = Perinecrotic zone; IT = Infiltrating Tumor; LE = Leading Edge. Differences among structures was assessed with the Kruskal-Wallis test.

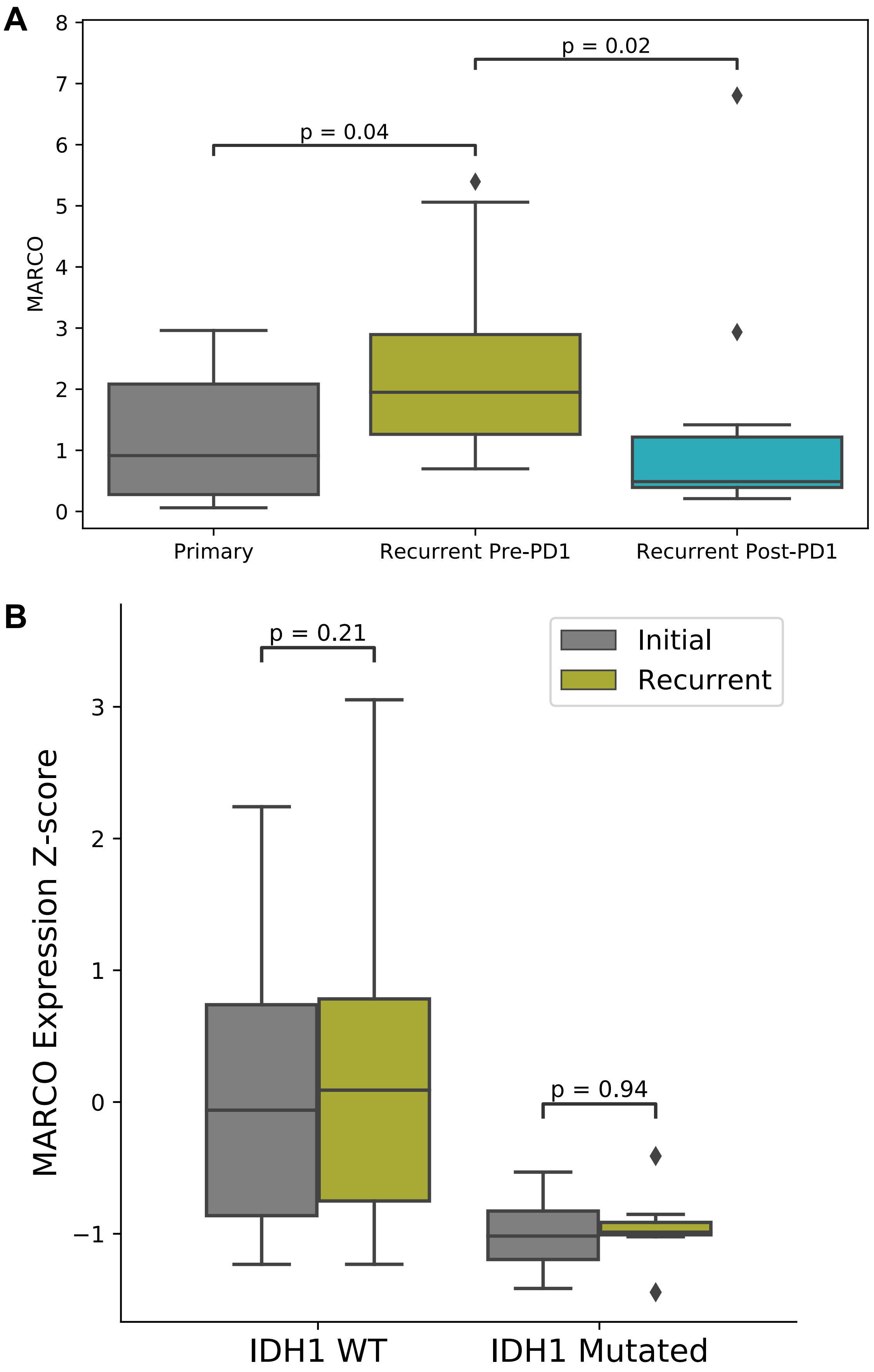

Fig. S10: Longitudinal MARCO expression under therapy. (A) Mean bulk MARCO expression within the longitudinal immunotherapy cohort of 17 PD1-treated patients, with responders and non-responders grouped together. **(B)** Mean bulk MARCO expression in a longitudinal cohort of 86 patients under standard therapy.

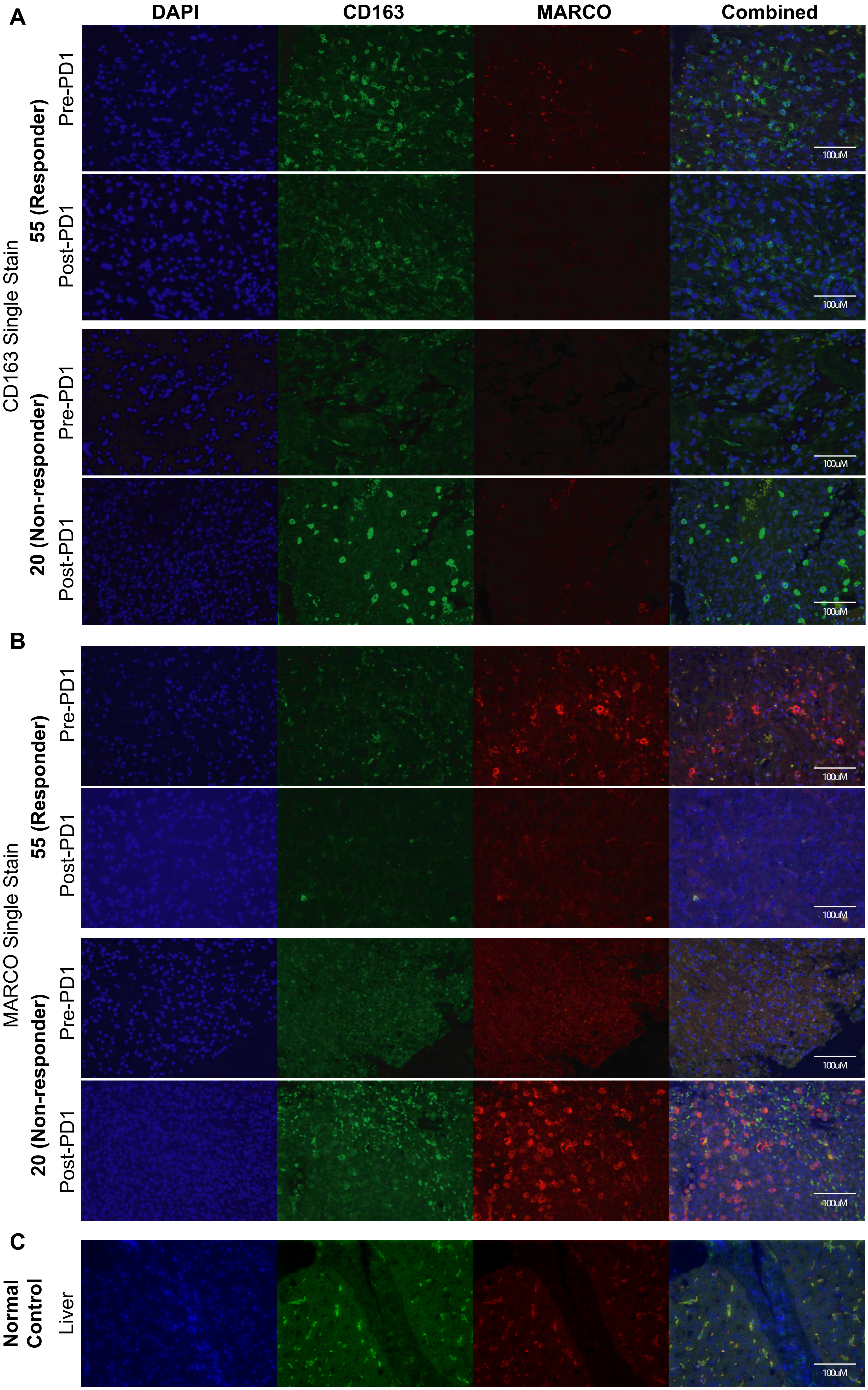

Fig. S11: Immunofluorescence imaging of MARCO+ macrophages before and after PD1 immunotherapy. Single-stain controls of **(A)** CD163 and **(B)** MARCO in the same samples (from patients 55 and 20) as imaged in Figure 4C. **(C)** Dual staining of normal liver control with the same markers.

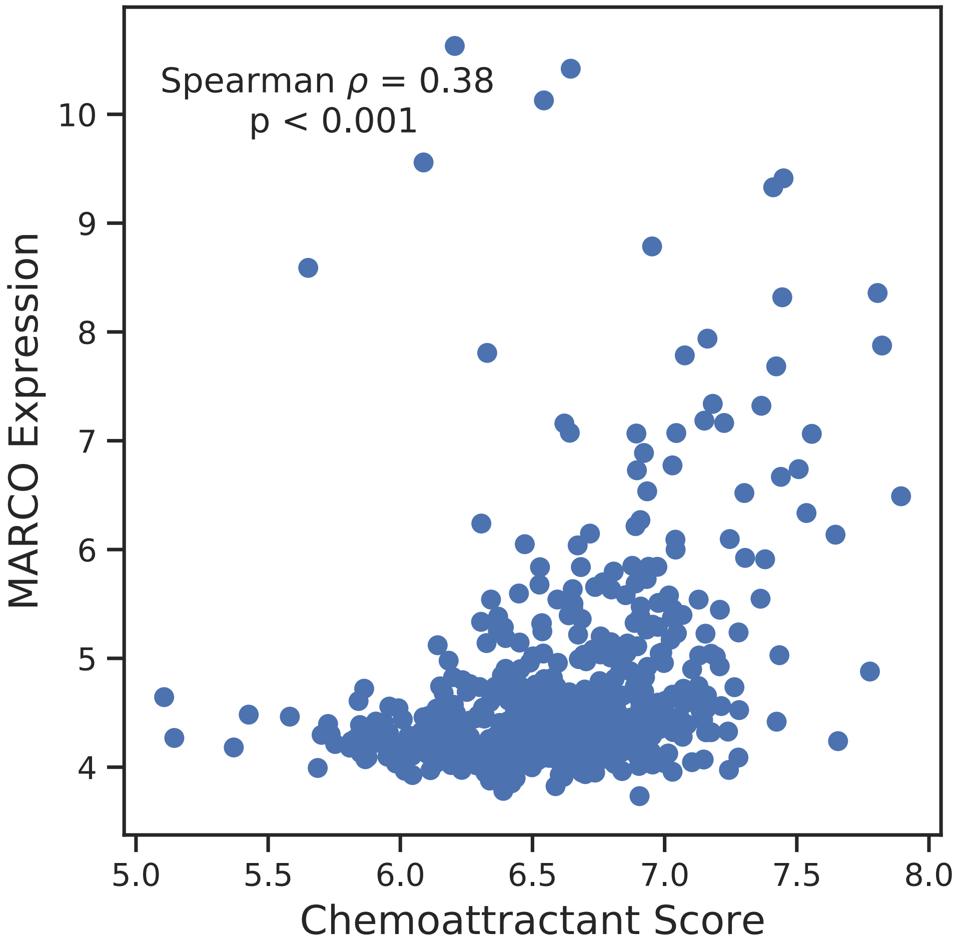

Fig. S12: Correlation of bulk MARCO expression with chemoattractant score in TCGA. Bulk transcriptomic validation of the correlation between MARCO and the chemoattractant score described in Fig. 5C.

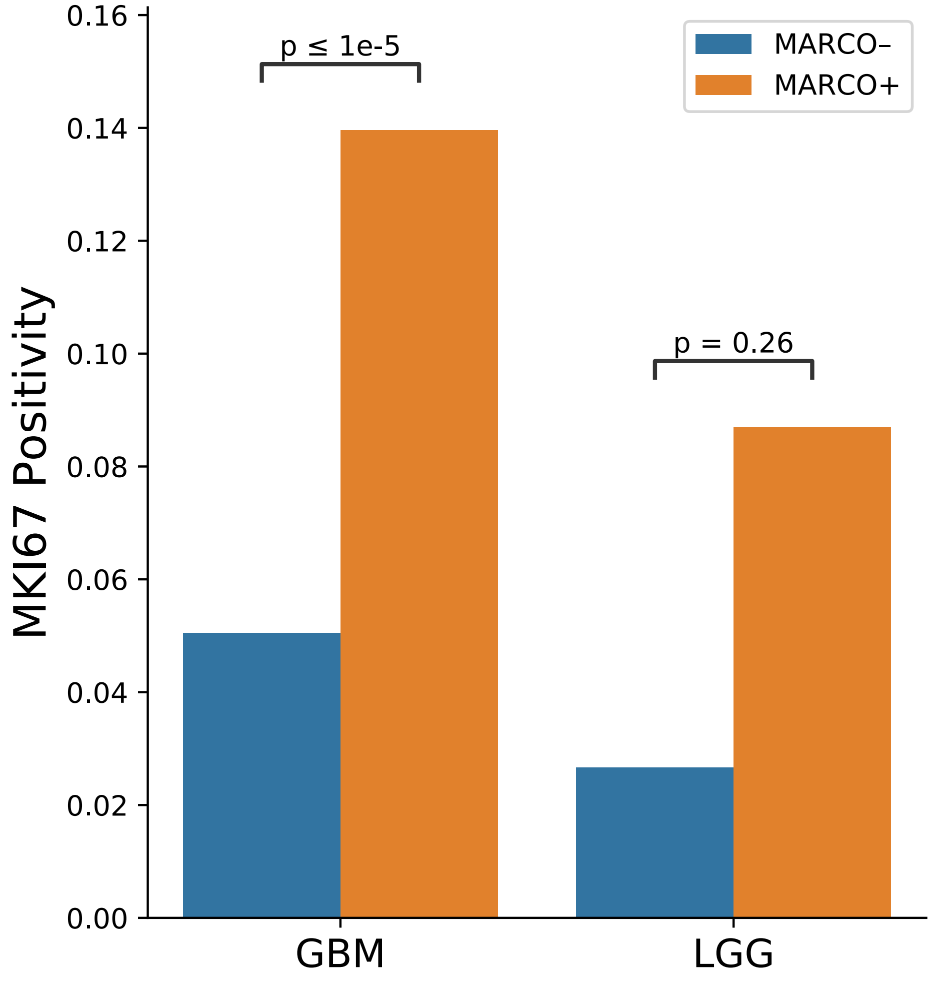

Fig. S13: Co-expression of proliferation markers in MARCO+ macrophages. The fraction of MARCO+ macrophages that co-express transcripts of MKI67 is shown for both GBM and LGG, compared to their MARCO– counterparts.
